# Toxic encephalopathy after an overdose of cocaine : a case serie

**DOI:** 10.1192/j.eurpsy.2023.1403

**Published:** 2023-07-19

**Authors:** R. Zouari, F. Nabli, D. Ben Mohamed, M. Z. Saeid, S. Ben Sassi

**Affiliations:** neurology, National institute of neurology mongi ben hmida, tunis, Tunis, Tunisia

## Abstract

**Introduction:**

cocaine is a widely used illegal drug, known for its fast ability to induce euphoria and arousal. However, cocaine exposure can contribute to several mental and physical effects. Cocaine induced brain damage can be divided into 3 mechanisms: direct effect leading to toxic encephalopathy, secondary to vascular damage causing vasculitis, stroke and vasospasm, and tertiary effect due to hypoxia through a cardiovascular collapse.

**Objectives:**

Here, we report 2 young men who developed a subacute encephalopathy with different clinical and radiological presentation after a cocaine overdose

**Methods:**

a case serie

**Results:**

we present two men aged respectively of 28 (P1) and 42 years-old (P2). Both had a history of alcohol consumption and toxicomania (mainly cocaine) during the past year. They manifested, 2 weeks following a cocaine overdose, with gait disorder and confusion. On examination, P1 was apathic and confused. He had a subcortical frontal syndrome with gait apraxia and grasping reflex, along with a quadri-pyramidal syndrome. While patient P2 developed a cognitive decline, parkinsonism with dystonic posture of the trunk and the right limbs, and a pseudobulbar syndrome. Brain MRI was performed in both patients and showed a bilateral multifocal leukoencephalopathy (P1) and the presence of bilateral hyper T2 and FLAIR weighted images affecting basal ganglia, the mesencephalon and the periventricular cerebral white matter. Cerebrospinal fluid (CSF) analysis showed no pleocytosis and normal proteinorrachia. Electroencephalogram was also normal. Infectious differential diagnosis including Human Immunodeficiency Virus (HIV) and syphilis were excluded and metabolic screening including copper analysis, serum and CSF lactate were normal. The urine toxic screening, performed 20 days following the drug overdose, was negative. Both patients were treated with benzodiazepine and fluids without significative improvement. They were discharged with major cognitive and motor impairment.

**Conclusions:**

cocaine toxicity is associated with high morbidity and mortality. Usually, cocaine abuse can lead to cardio-vascular, pulmonary and nervous complication. Neuropsychiatric sequelae are uncommon with less recovery potential. To date, there is no drug to prevent or cure cocaine addiction. The key is to educate the patient when he or she presents to the emergency department. Patients should be urged to seek drug counseling.

**Disclosure of Interest:**

None Declared

